# RORγt^+^ Treg to Th17 ratios correlate with susceptibility to *Giardia* infection

**DOI:** 10.1038/s41598-019-56416-9

**Published:** 2019-12-30

**Authors:** Ivet A. Yordanova, Alba Cortés, Christian Klotz, Anja A. Kühl, Markus M. Heimesaat, Cinzia Cantacessi, Susanne Hartmann, Sebastian Rausch

**Affiliations:** 1 Institute of Immunology, Centre for Infection Medicine, Freie Universität Berlin, Berlin, Germany; 2 Department of Veterinary Medicine, University of Cambridge, Cambridge, United Kingdom; 3 Unit 16 Mycotic and Parasitic Agents and Mycobacteria, Department of Infectious Diseases, Robert Koch-Institute, Berlin, Germany; 4 iPATH.Berlin, Core Unit for Immunopathology for Experimental Models, Charité - University Medicine Berlin, corporate member of Freie Universität Berlin, Humboldt-Universität zu Berlin, and Berlin Institute of Health, Berlin, Germany; 5 Institute of Microbiology, Infectious Diseases and Immunology Charité - University Medicine Berlin, corporate member of Freie Universität Berlin, Humboldt-Universität zu Berlin, and Berlin Institute of Health, Berlin, Germany

**Keywords:** Cellular immunity, Mucosal immunology, Mucosal immunology, Parasite host response, Parasite host response

## Abstract

Infections with *Giardia* are among the most common causes of food and water-borne diarrheal disease worldwide. Here, we investigated Th17, Treg and IgA responses, and alterations in gut microbiota in two mouse lines with varying susceptibility to *Giardia muris* infection. Infected BALB/c mice shed significantly more cysts compared with C57BL/6 mice. Impaired control of infection in BALB/c mice was associated with lower Th17 activity and lower IgA levels compared with C57BL/6 mice. The limited metabolic activity, proliferation and cytokine production of Th17 cells in BALB/c mice was associated with higher proportions of intestinal Foxp3^+^RORγt^+^ regulatory T cells and BALB/c mice developed increased RORγt^+^ Treg:Th17 ratios in response to *G. muris* infection. Furthermore, *G. muris* colonization led to a significantly reduced evenness in the gut microbial communities of BALB/c mice. Our data indicate that differential susceptibility to *Giardia* infections may be related to RORγt^+^ Treg controlling Th17 activity and that changes in the microbiota composition upon *Giardia* infection partially depend on the host background.

## Introduction

Infections with the intestinal protozoan parasite *Giardia lamblia* remain a highly prevalent cause of food- and water-borne diarrheal disease across the world. Recent data indicate that over 183 million cases of giardiasis occur annually across the globe, with a disease burden of almost 172,000 Disability Adjusted Life Years (DALYs)^1^. *G. lamblia* accounts for ~35–37% of water-borne disease outbreaks and is estimated to cause 0.5–5.4% of cases of diarrhea in children under 5 years of age in both high- and low-income countries^2,3^. *Giardia* trophozoites are typically non-invasive and preferentially colonize the upper small intestinal tract, where they attach in dense foci to the epithelial cell layer, a mechanism thought to contribute to localized and generally mild immunopathology^4,5^. Giardiasis commonly causes few signs of intestinal inflammation^6^, however some patients do develop diverse clinical manifestations, such as chronic diarrhea, abdominal pain, fatigue and malabsorption^6^. Studies surveying *Giardia* infection intensity in human patients indicate a wide variation in fecal cyst shedding rates in children^7^. Similarly, studies in dairy calves infected with *G. lamblia* indicate significant individual variations in fecal cyst shedding^8^. The potential factors influencing such differences in parasite loads, overt immunopathology or the development of clinical symptoms are yet to be characterised.

*Ex vivo* stimulation of blood cells, small intestinal lamina propria (siLP) lymphocytes or intestinal epithelial lymphocytes from *G. lamblia*-infected patients leads to the production of a number of pro-inflammatory cytokines, including IL-17A^9,10^. Recent work in experimental mouse models has demonstrated that Th17 cells and IL-17A production are crucial players in the efficient control of *Giardia* infection^11–13^. IL-17A typically drives neutrophil recruitment, antimicrobial peptide secretion and supports the expression of the polymeric Ig receptor (pIgR) by intestinal epithelial cells, which is necessary for IgA transport into the intestinal lumen^11,13,14^. IgA secretion represents another key immune protective mechanism against *Giardia*, as mice deficient in B cells, IgA or pIgR fail to control infection with *G. lamblia* or *G. muris*, the species adapted to the murine host^11,15,16^.

The high prevalence of asymptomatic *Giardia* infections and the minimal immunopathology commonly observed during experimental giardiasis suggests that immune-regulatory mechanisms may participate in controlling the pro-inflammatory potential of Th17 cells, possibly at the expense of efficient elimination of infection^8^. Regulatory T cells (Treg) expressing the transcription factor Foxp3 are pivotal for the maintenance of homeostasis and regulation of overt inflammatory processes^17^. In recent years it was shown that Foxp3^+^ Treg adopt functional specialization by expressing the transcription factors T-bet, GATA-3 or RORγt associated with the Th1, Th2 and Th17 effector lineages, respectively. These effector-like Treg populations were shown to efficiently co-localize with the respective effector T cell subsets and to constrain inflammatory responses^18–20^. Hence, distinct subsets of Treg are a key factor balancing immune responses at mucosal surfaces^21^. RORγt^+^ Treg have been characterized as peripherally-induced and highly enriched in the small intestinal and colonic lamina propria^20,22^. Their differentiation is primarily dependent on commensal microbiota signaling and their absence leads to elevated IL-17A expression by intestinal T cells^20,23,24^.

Intestinal Treg heterogeneity and associations between Treg phenotypes and Th17 cell activity have not been addressed in the context of *Giardia* infections to date. Here, we assessed Th17, Treg and IgA responses and the microbiota composition in two mouse lines with differential susceptibility to *G. muris* infection. We found that BALB/c mice releasing moderately higher cyst numbers display poor Th17 activity, increased Foxp3^+^RORγt^+^ Treg to Th17 cell ratios, limited IgA production and more pronounced changes in the microbiota structure compared to C57BL/6 mice, which restrict *Giardia* replication more readily.

## Results

### Differential control of *Giardia* infection is associated with Th17 activity

**Figure 1:**
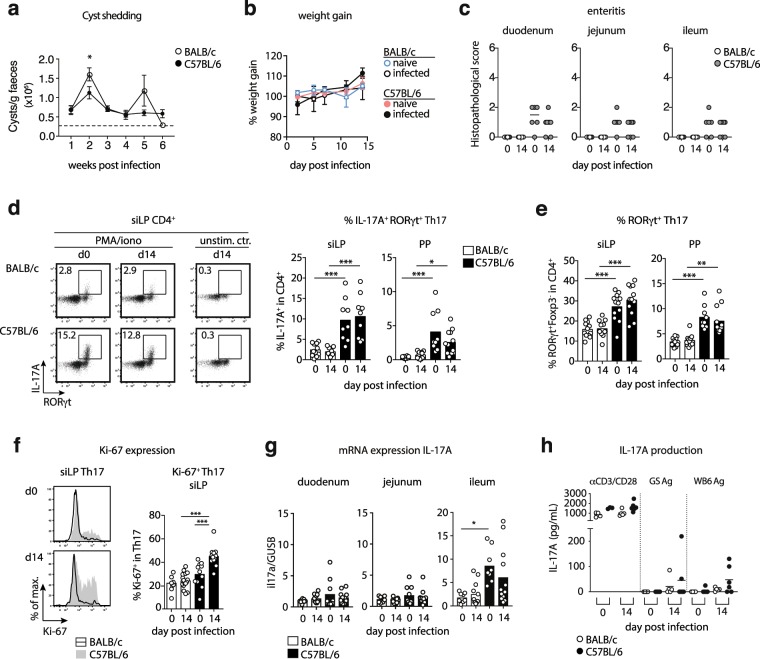
Differential Th17 activity at steady state and during *G. muris* infection in BALB/c and C57BL/6 mice. Mice were orally infected with 1,000 *G. muris* cysts and surveyed for cyst excretion for six weeks and for intestinal Th17 cell activity 14 days post-infection. (**a**) Cyst shedding dynamics over the course of six weeks of *G. muris* infection in BALB/c and C57BL/6 mice. (**b**) Weight gain (relative to starting weight) of naïve and *G. muris*-infected BALB/c and C57BL/6 mice during a 14 day infection period. (**c**) Small intestinal enteritis score in duodenum, jejunum and ileum of naïve and *G. muris*-infected BALB/c and C57BL/6 mice. (**d**) Representative FACS plots of RORγt and IL-17A expression within CD4^+^ T cells in siLP of naïve and infected BALB/c and C57BL/6 mice following PMA/Ionomycin stimulation and frequencies of IL-17A^+^RORγt^+^ cells in CD4^+^ T cells in siLP and PP. (**e**) Frequencies of RORγt^+^Foxp3^-^ Th17 cells in CD4^+^ cells in siLP and PP of naïve and infected BALB/c and C57BL/6 mice. (**f**) Ki-67 expression in RORγt^+^ Th17 cells in siLP. (**g**) IL-17A expression relative to the housekeeping gene β-glucuronidase (GUSB) as determined in small intestinal tissue samples. (**h**) Parasite-specific IL-17A expression by splenocytes of naïve and infected BALB/c and C57BL/6 mice stimulated with anti-CD3/CD28 antibodies or *G. lamblia* trophozoite GS or WB6 antigen. Signals from unstimulated control wells (all <36 pg/ml) were subtracted. Data in a, d, e, f and g are pooled from two-three independent experiments, with n = 3–5 mice/group. Weight gain was monitored once. Data in c and h are pooled from two independent experiments each performed with n = 3–4. Statistical analysis was done using Kruskal-Wallis test combined with Dunn’s or Tukey’s multiple comparison test (a-d) or unpaired t test (e). *p < 0.05, **p < 0.01, ***p < 0.001.

Over the course of 6 weeks, BALB/c mice displayed higher fluctuations in cyst shedding and shed significantly more cysts in the second week after infection compared with C57BL/6 mice. (Fig. 1a). The moderately higher cyst numbers released by infected BALB/c mice were not associated with changes in body weight development (Fig. 1b). Furthermore, BALB/c mice displayed no signs of intestinal inflammation. In contrast, enteritis scores were slightly elevated in C57BL/6 mice irrespective of the infection status, which was due to mild leukocyte infiltrations and/or slight blunting of villi (Fig. 1c). Previous studies demonstrated that the efficient control of *Giardia* infection depends on Th17 cells secreting IL-17A^11,12^. Asking if the moderate difference in susceptibility to *Giardia* infection correlated with the activity of Th17 cells, we surveyed RORγt and IL-17A expression by CD4^+^ T cells in BALB/c and C57BL/6 mice at steady state and two weeks post *G. muris* infection. C57BL/6 mice harbored higher frequencies of Th17 cells expressing IL-17A and RORγt within CD4^+^ lymphocytes isolated from the small intestinal lamina propria (siLP) and Peyer’s patches (PP) compared to BALB/c mice (Fig. 1d,e). Of note, the frequencies of small intestinal CD4^+^ effector cells expressing RORγt^+^ and IL-17A^+^ remained stable upon infection in both mouse lines (Fig. 1d,e) despite significantly higher proportions of siLP Th17 cells expressing the proliferation marker Ki-67 in infected C57BL/6 mice (Fig. 1f). We hence assessed the expression of the tissue retention markers CD69 and CD103 by small intestinal Th17 cells to see if this discrepancy might be due to recirculation of Th17 cells from gut tissue in infected C57BL/6 mice. However, small intestinal Th17 cells of both mouse lines similarly expressed CD69 and CD103 at steady state and upon infection (Suppl. Figure 1a,b).

Next, we investigated if the stronger IL-17A expression detected in small intestinal CD4^+^ of C57BL/6 mice was reflected *in vivo* by quantifying IL-17A gene transcription in the small intestine. IL-17A transcript levels were significantly higher in the ileum of naive C57BL/6 compared with naïve BALB/c mice. However, corroborating the unaltered IL-17A protein expression detected in intestinal CD4^+^ T cells upon restimulation, IL-17A transcription in duodenum, jejunum and ileum was not augmented two weeks after *G. muris* infection (Fig. 1g).

To test if differences in parasite-specific Th17 responses were associated with the differential control of *Giardia* replication in the two mouse lines, we stimulated cells isolated from spleen, mLN and PP of naïve and *G. muris*-infected BALB/c and C57BL/6 mice with trophozoite antigen derived from the two *G. lamblia* strains GS (assemblage B) and WB6 (assemblage A) or with anti-CD3/CD28 antibodies as a positive control. IL-17A responses triggered by anti-CD3/CD28 antibodies did not differ depending on the infection status in cultures of spleen, mLN or PP cells (Fig. 1h and data not shown). Parasite-specific IL-17A responses to both types of antigen were detected in spleen cell cultures of several infected C57BL/6 mice, while the corresponding cultures of most infected BALB/c mice responded poorly to the antigens (Fig. 1h). Interestingly, IL-17A production in response to the parasite antigens was only detected with spleen cells, whereas parallel cultures of mLN and PP cells did not react to parasite antigens (data not shown).

Taken together, these data indicate that lower cyst shedding by *G. muris* infected C57BL/6 mice relates to constitutively higher frequencies of intestinal Th17 cells and the more rapid development of parasite-specific IL-17A responses to *Giardia* infection compared to BALB/c mice

### Restricted Th17 responses correlate with elevated frequencies of intestinal RORγt^+^ Treg

**Figure 2:**
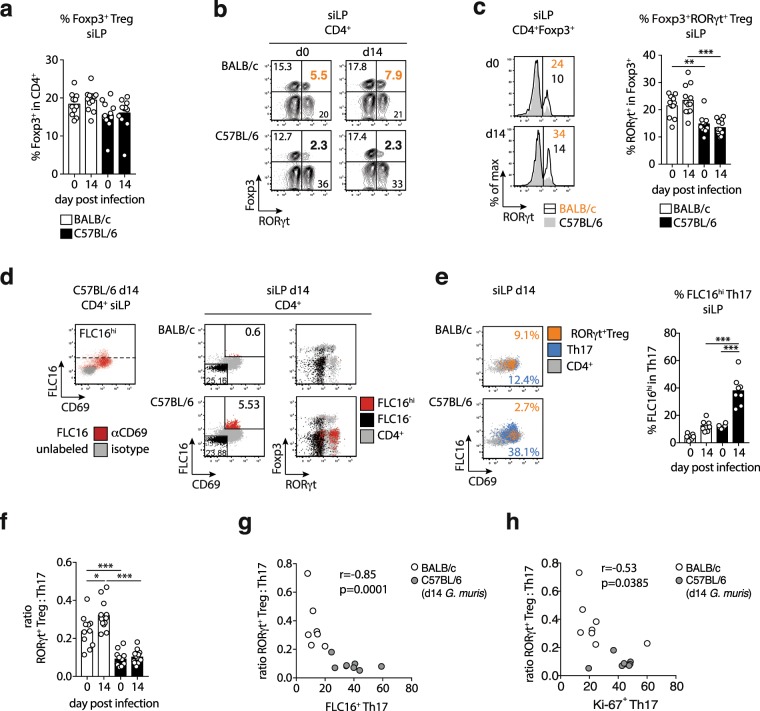
High Foxp3^+^RORγt^+^ Treg proportions correlate with poor intestinal Th17 activity in BALB/c mice. (**a**) Frequencies of Foxp3^+^ Treg in CD4^+^ cells isolated from the siLP of naïve and *G. muris* infected (day 14) BALB/c and C57BL/6 mice. (**b**) Representative FACS plots of RORγt and Foxp3 expression by small intestinal CD4^+^ T cells. Bold numbers report frequencies of Foxp3^+^RORγt^+^ Treg in BALB/c (orange) and C57BL/6 mice (black). (**c**) Representative histogram plots of RORγt expression in Foxp3^+^ Treg and frequencies of RORγt^+^ cells in the Foxp3^+^ Treg population isolated from siLP of naïve and infected BALB/c and C57BL/6 mice. (**d**) Uptake of fluorescently labelled palmitate (Bodipy FLC16) by CD4^+^ T cells isolated from the small intestine of mice infected with *G. muris* for two weeks. Left: representative overlays of unlabeled and labeled cells isolated from a C57BL/6 mouse at day 14 post infection. The demarcation of FL16^high^ cells is indicated by the dashed line. Centre: gating of FL16^high/low^ cells in CD4^+^ T cells isolated from BALB/c (B/c) and C57BL/6 (BL/6) mice at day 14 post infection. Right: expression profiles of Foxp3 and RORγt of FL16^high/low^ cells overlayed onto the total CD4^+^ T cell population. (**e**) Representative overlays of Foxp3^+^RORγt^+^ Treg, RORγt^+^ Th17 and total CD4^+^ cells. Numbers report frequencies of RORγt^+^ Treg (orange) and Th17 cells (blue) in CD4^+^ T cells. The frequencies of FLC16^hi^ cells within CD4^+^RORγt^+^ Th17 cells isolated from the small intestine are reported in the bar graph. (**f**) Ratio of RORγt^+^ Treg to Th17 cells in small intestinal CD4^+^ cells. (**g**,**h**) Correlation between RORγt^+^ Treg to Th17 cell ratios and (g) the frequency of FLC16^hi^ Th17 cells or (h) Ki-67^+^ Th17 cells in small intestinal isolates of *G. muris*-infected BALB/c and C57BL/6 mice. Data are pooled from three (a,c,f) and two (e,g,h) independent experiments with n = 3–5/group. Statistical analysis was done using Kruskal-Wallis test combined with Dunn’s or Tukey’s multiple comparison test (a–f) and Spearman correlation test (g,h). *p < 0.05, **p < 0.01, ***p < 0.001.

The poor Th17 activity in BALB/c compared with C57BL/6 mice at steady state and during *G. muris* infection prompted the question if this was associated with differences in the regulatory T cell (Treg) compartment. Frequencies of Foxp3^+^ Treg within the CD4^+^ T cell population were similar in the small intestine of both mouse lines and did not change during infection in BALB/c and C57BL/6 mice (Fig. 2a). Confirming the flow cytometry data, similar numbers of CD4^+^ T cells and CD4^+^Foxp3^+^ Treg were detected for all groups in cross sections of duodenum, jejunum and ileum (Fig. S1c,d). Peripherally induced Foxp3^+^Helios^−^ Treg cells co-expressing RORγt have previously been reported to display superior suppressive activity compared to conventional RORγt^−^ Treg^22,25^. We found that Foxp3^+^ Treg isolated from the small intestine of BALB/c mice comprised significantly more RORγt^+^ cells compared with Treg derived from C57BL/6 mice (Fig. 2b,c). In line with previous reports^22,25^, RORγt^+^ Treg largely lacked Helios expression, indicating their extra-thymic origin (Fig. S1e). Next, we surveyed the uptake of fluorescently-labelled palmitate (Bodipy FL16) as a measure of cellular metabolic activity. In both infected mouse lines, RORγt^−^ and RORγt^+^Foxp3^+^ Treg acquired higher levels of palmitate compared to Foxp3^−^RORγt^−^CD4^+^ cells (Fig. S1f). CD4^+^ T cells isolated from the small intestine of *G. muris* infected C57BL/6 mice, but not BALB/c mice, comprised a brightly FL16-labeled population, which predominantly originated from RORγt^+^ Th17 cells expressing the early T cell activation marker CD69 (Fig. 2d). *G. muris* infection led to a significant increase in palmitate uptake by Th17 cells in infected C57BL/6 mice compared with naive controls and with infected BALB/c mice (Fig. 2e). Furthermore, BALB/c mice displayed significantly higher ratios of RORγt^+^ Treg to Th17 cells at steady state compared with C57BL/6 mice and this ratio increased further in response to *G. muris* infection in BALB/c, but not C57BL/6 mice (Fig. 2f). Consequently, we detected a significant negative correlation between the ratio of RORγt^+^ Treg to Th17 cells and the metabolic activity, as well as the proliferative response of intestinal Th17 cells (Fig. 2g,h). In conclusion, *G. muris* infection induces a significant shift in favor of RORγt^+^ Treg in BALB/c, but not C57BL/6 mice. In addition, high ratios of RORγt^+^ Treg to Th17 cells correlate with poor activity in local Th17 cells in *Giardia* infected mice.

### Poor ILC3 activity in BALB/c mice at steady state and during *G. muris* infection

**Figure 3:**
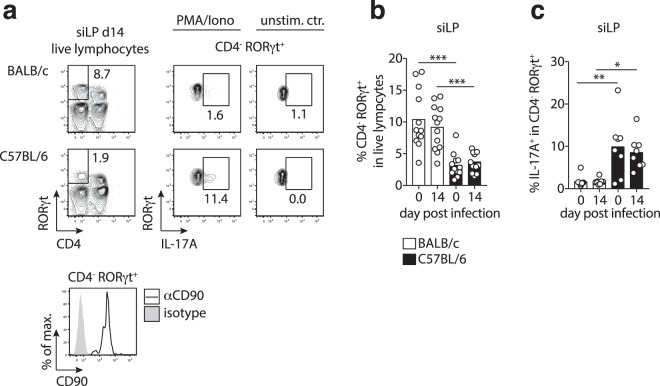
Elevated frequencies, but poor activity of ILC3 in the small intestine of BALB/c mice. (**a**) Representative FACS plots of CD4 and RORγt expression by small intestinal cells in *G. muris*-infected BALB/c and C57BL/6 mice 14 days post-infection (left panel) and of IL-17A expression by CD4^-^RORγt^+^ cells in naïve and infected BALB/c and C57BL/6 mice (right panel). The histogram plot reports CD90 expression by CD4^-^RORγt^+^ cells. (**b**) Frequencies of CD4^-^RORγt^+^ cells in siLP cells. (**c**) Frequencies of IL-17A^+^ cells in CD4^-^RORγt^+^ cells after PMA/ionomycin stimulation. Data are pooled from three (b) or two (c) independent experiments with n = 3–5/group. Statistical analysis was done using Kruskal-Wallis test combined with Dunn’s multiple comparison test. *p < 0.05, **p < 0.01, ***p < 0.001. Further information on the phenotype of CD4^-^RORγt^+^ cells is available in Supplementary Fig. 2d–g.

A previous study reported the increased expression of IL-17A by innate immune cells along with CD4^+^ T cells during *G. muris* infection in C57BL/6 mice^11^. We hence asked if BALB/c and C57BL/6 mice differed in IL-17A responses by non-CD4^+^ T cells. Contrasting the higher frequencies of Th17 cell in the small intestines of C57BL/6 mice, we detected significantly more CD90^+^CD4^−^RORγt^+^ group 3 innate lymphoid cells (ILC3) in naïve and infected BALB/c mice (Fig. 3a,b). However, as seen for Th17 cells, small intestinal ILC3 of naïve and infected BALB/c mice poorly produced IL-17A following *ex vivo* stimulation compared with those from C57BL/6 mice (Fig. 3a,c). ILC3 from PP mirrored this pattern of IL-17A responses (Fig. S2a), whereas IL-17A production by mLN-derived ILC3 was similar in both mouse lines (Fig. S2b,c). Thus, higher *G. muris* cyst excretion by BALB/c mice coincides with poor IL-17A production by Th17 cells and ILC3 in the local vicinity of the parasite.

### Systemic and local IgA levels mirror small intestinal Th17 activity at steady state and during *Giardia* infection

**Figure 4:**
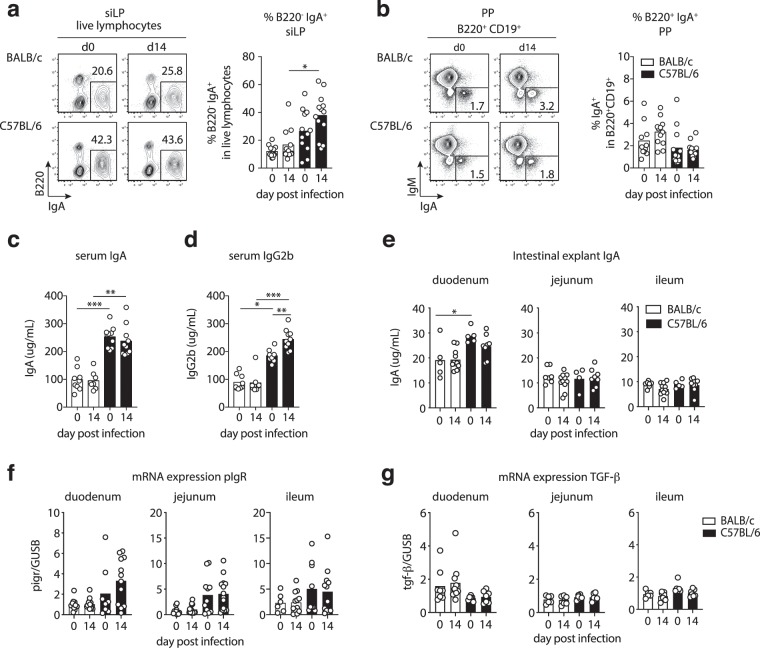
C57BL/6 mice display elevated small intestinal IgA^+^ plasma cells and serum IgA/IgG2b levels compared with BALB/c mice. (**a**) Representative FACS plots of IgA and B220 expression by siLP cells. Frequencies of B220^-^IgA^+^ plasma cells in siLP isolates are reported in the bar graph. (**b**) Representative FACS plots of IgA and IgM expression by B220^+^ B cells and frequencies of B220^+^IgA^+^ B cells in small intestinal Peyer’s patches (PP). (**c**,**d**) Serum levels of (**c**) IgA and (**d**) IgG2b antibodies. (**e**) IgA antibody levels in small intestinal tissue explant supernatants. (**f**,**g**) pIgR and TGF-β mRNA expression relative to GUSB determined in small intestinal tissue samples. Data are pooled from three (a–d) and two (e–g) independent experiments with n = 3–5/group. Statistical analysis was done using Kruskal-Wallis test combined with Dunn’s multiple comparison test. *p < 0.05, **p < 0.01, ***p < 0.001.

Intestinal IgA is a key host protective factor in giardiasis^11,15,16^. We hence investigated if differential parasite control by C57BL/6 compared with BALB/c mice coincided with differences in their local and systemic antibody responses. We therefore assessed the frequencies of B220^−^IgA^+^ plasma cells (PC) in siLP and B220^+^CD19^+^IgA^+^ B cells in PP, as well as serum and intestinal IgA titers. Indeed, siLP isolates of C57BL/6 mice comprised higher frequencies of B220^−^IgA^+^ plasma cells compared with BALB/c cell isolates, reaching significance two weeks post-*G. muris* infection (Fig. 4a). In contrast, similar frequencies of IgA^+^ B cells were present in PP of both mouse lines at steady state and following infection (Fig. 4b). Serum IgA levels were similar at steady state and two weeks post infection, however, both naive and infected C57BL/6 mice had significanty more IgA in serum compared to BALB/c mice (Fig. 4c). Higher serum IgA levels were accompagnied by significantly more IgG2b in serum of naïve C57BL/6 compared to BALB/c mice and IgG2b levels increased further in response to *G. muris* infection in the C57BL/6, but not the BALB/c line (Fig. 4d). To estimate possible differences in secretory IgA transport into the intestinal lumen, we quantified IgA released by intestinal tissue explants and assessed pIgR transcript levels in small intestinal tissue. Duodenal explants isolated from naïve CD57BL/6 mice released significantly higher amounts of IgA compared to tissue from BALB/c mice and a similar trend was maintained during infection (Fig. 4e). IgA levels secreted by jejunum and ileum explants were similar for all groups (Fig. 4e). In trend, pIgR transcript levels were increased in duodenum, jejunum and ileum of C57BL/6 compared with BALB/c mice (Fig. 4f). As both IgA and IgG2b class switching are supported by the cytokine TGF-β^26,27^, we surveyed TGF-β mRNA and protein levels in intestinal samples of BALB/c and C57BL/6 mice and found no evidence for differential TGF-β transcription or TGF-β protein levels depending on the host background and infection status (Fig. 4g and data not shown). Thus, more efficient parasite control by C57BL/6 mice coincided with a more prominent population of intestinal IgA^+^ PC and systemically elevated IgA and IgG2b levels at steady state and during *G. muris* infection.

### Alterations in microbiota structure resulting from *G. muris* infection differ depending on the host genetic background

The gut microbiota is pivotal for the development of intestinal Th17 and IgA responses which, in turn, regulate the composition of the microbial community^28,29^. We hence asked if the differential Th17 and IgA responses detected in BALB/c compared with C57BL/6 mice under homeostasis and upon *G. muris* infection were associated with distinct microbiota signatures. Comparing the fecal microbiome of naïve BALB/c and C57BL/6 mice, we detected differences in the microbiota structure between the two mouse lines (Fig. S3a,b). In particular, microbial beta diversity (i.e., differences in microbial species composition between groups) measured by ANOSIM on Bray-Curtis dissimilarities, was significantly lower in C57BL/6 than in BALB/c mice (p = 0.007; Fig. S3c). Furthermore, whilst no significant differences were detected in overall alpha diversity (measured through the Shannon index) and microbial richness (i.e., the number of species composing a given microbial community), a significantly lower bacterial evenness (i.e., the relative abundance of each microbial species within a given microbial community; p = 0.035, ANOVA) was observed in C57BL/6 compared with BALB/c mice (Fig. S3d). Comparison of individual microbial taxa abundances by Linear discriminant analysis Effect Size (LEfSe) and negative binomial distribution (DESeq. 2) revealed significant differences (from phylum to genus level) between the two mouse lines, including several families/genera within the order Clostridiales (Table S1, S2).

**Figure 5:**
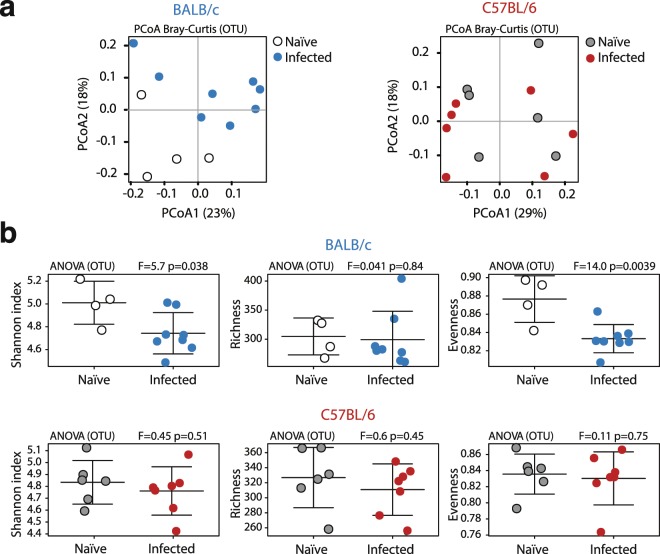
Principle coordinates analysis (PCoA) and diversity of the fecal microbiota during *G. muris* infection. (**a**) Unsupervised Principal Coordinates Analysis (PCoA, on Bray-Curtis dissimilarities) of the fecal microbiota composition of naïve and infected (day 14) BALB/c and C57BL/6 mice. (**b**) Alpha diversity (measured by the Shannon index), species richness and evenness of the fecal microbiota of naïve and *G. muris*-infected BALB/c and C57BL/6 mice. Data are pooled from two independent experiments with n = 2–4 mice/group.

Analysis of the microbiome composition of *G. muris*-infected BALB/c and C57BL/6 mice in comparison with their respective uninfected controls revealed significant alterations of the gut microbial community of infected BALB/c, but not C57BL/6 mice, as detected by unsupervised Principal Coordinates Analysis (PCoA, on Bray-Curtis dissimilarities) (Fig. 5a). The observed alterations in gut microbial community profiles of infected BALB/c mice were associated with a decrease in overall alpha diversity (measured through Shannon index; p = 0.038, ANOVA), which was driven by a significant reduction in microbial evenness (p = 0.0039, ANOVA), whilst species richness remained unaltered (Fig. 5b). In contrast, no alterations in alpha diversity were observed in the microbiota of C57BL/6 mice upon infection (Fig. 5b). Microbial beta diversity measured by ANOSIM on Bray-Curtis dissimilarities did not reveal significant differences between infected and uninfected mice of either mouse lines (Fig. S4).

**Figure 6:**
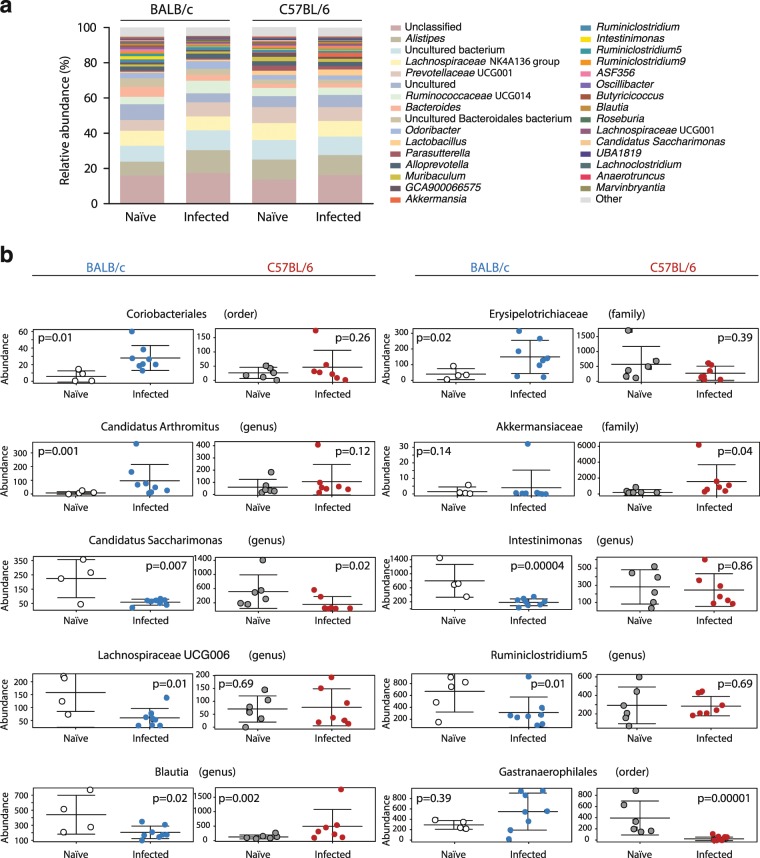
Populations of gut bacteria impacted by *G. muris* infection differ between BALB/c and C57BL/6 mice. (**a**) Microbial composition, at genus level, of naïve and *G. muris*-infected (day 14) mice of each line. Genera making up <0.5% of the overall microbiota are grouped under ‘others’. (**b**) Abundances of taxa varying significantly between naïve/infected BALB/c and/or naïve/infected C57BL/6 mice. Data are pooled from two independent experiments with n = 2–4 mice/group.

Importantly, populations of gut bacteria significantly impacted by *G. muris* colonization according to DESeq. 2 and LEfSe varied between the two mouse lines (Fig. 6a, Tables S3, S4). *G. muris* infection led to an increase of Coriobacteriales (fold change = 4.37, p = 0.013) and a minor expansion of *Erysipelotrichaceae* (fold change = 3.21, p = 0.034) in BALB/c, but not in C57BL/6 mice (Fig. 6b). Surprisingly, segmented filamentous bacteria (SFB, genus *Candidatus Arthromitus*) were identified as the most prominently expanding taxon in infected BALB/c mice (Fold change = 10.38, p = 0.001) (Fig. 6b), whereas infected C57BL/6 mice displayed a significant increase in the abundance of the *Akkermansiaceae* family (fold change = 8.72, p = 0.043) (Fig. 6b). The abundance of *Candidatus Saccharimonas* was significantly reduced in both mouse lines upon *G. muris* infection (fold change = −4.45/−3.69, p = 0.007/0.022) (Fig. 6b). Furthermore, significant alterations in the composition of the order of Clostridiales were detected in infected BALB/c and C57BL/6 mice. Specifically, we detected a reduction in populations of the genera *Intestinimonas* (fold change = −5.31, p = 0.00004), *Lachnospiraceae* UCG006 (fold change = −3.43, p = 0.01), *Ruminiclostridium* 5 (fold change −2.92, p = 0.01) and *Blautia* (fold change = −2.68, p = 0.02) in infected BALB/c mice, whereas *Blautia* expanded in infected C57BL/6 mice (fold change = 4.21, p = 0,002) (Fig. 6b). Finally, infected C57BL/6 mice displayed a highly significant decrease in the abundance of Gastranaerophilales (Fold change = −17.96, p = 0.00001; DESeq. 2) (Fig. 6b). LEfSe analysis (Table S4) supported the results obtained by DESeq. 2 (except for *Blautia*) and revealed that, within the order Coriobacteriales, the family *Eggerthellaceae*, and the genus *Enterorhabdus* in particular, were significantly more abundant in the gut of infected BALB/c mice, whilst the genus *Akkermansia* was associated with *G. muris* infection in C57BL/6 mice (Table S4).

**Figure 7:**
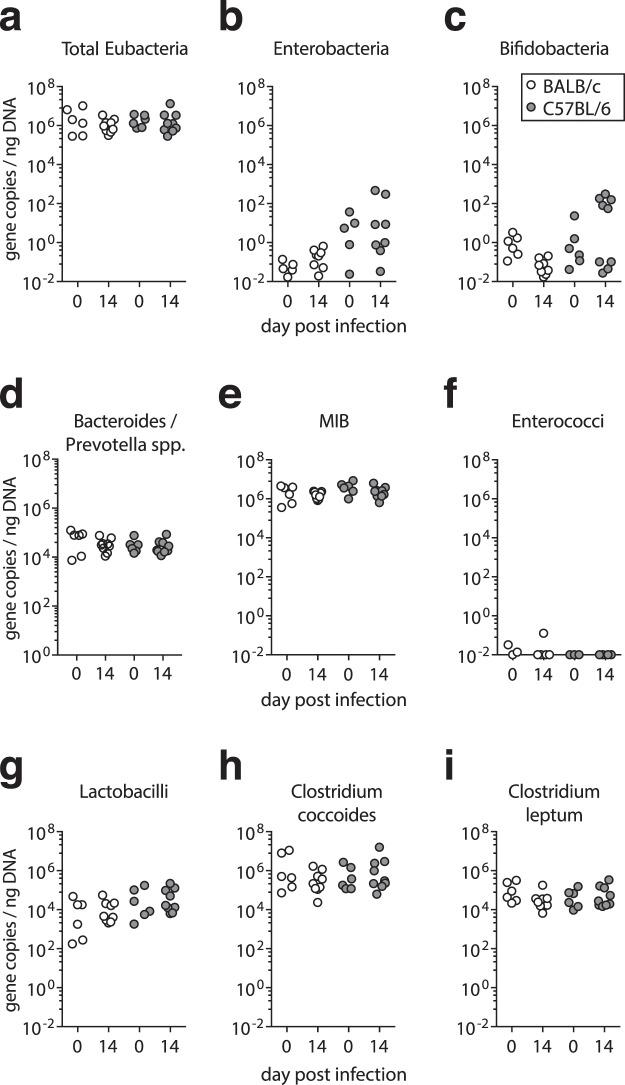
Abundance of the main bacterial groups present in the murine intestinal microbiota detected by qPCR in naïve and *G. muris* infected BALB/c and C57BL/6 mice. Fecal gene numbers of the main bacterial groups abundant in the murine intestinal microbiota detected by qPCR in naïve and *G. muris* infected (day 14) BALB/c and C57BL/6 mice. Individual fecal loads of (**a**) total Eubacteria, (**b**) Enterobacteria, (**c**) Bifidobacteria, (**d**) *Bacterides*/*Prevotella* spp., (**e**) Mouse Intestinal Bacteroides (MIB), (**f**) Enterococci, (**g**) Lactobacilli, (**h**) *Clostridium coccoides* and (**i**) *Clostridium leptum*. Results are expressed as 16 R rRNA gene copy numbers per ng DNA. Data are pooled from two independent experiments with n = 3–5/group.

Further 16S rRNA-based qPCR analysis of the fecal microbiota composition showed that the total bacterial load remained stable in both mouse lines upon *G. muris* infections (Fig. 7a) and confirmed that the abundance of members of Proteobacteria (Enterobacteria, Fig. 7b), Actinobacteria (Bifidobacteria, Fig. 7c), Bacteroidetes (*Bacteroides*/*Prevotella* spp. and Mouse Intestinal Bacteroidetes, MIB; Fig. 7d,e) and Firmicutes (*Enterococci, Lactobacilli, Clostridium coccoides*/*leptum* groups, Fig. 7f–i) remained unaltered during *G. muris* infection and were comparable in abundance between naive and infected BALB/c and C57BL/6 mice.

## Discussion

Our study shows that mice with a different genetic background display differential susceptibility towards *G. muris* colonization. In accordance with previous reports of prolonged and elevated *G. muris* cysts excretion and higher numbers of *G. lamblia* trophozoites in infected BALB/c compared with C57BL/6 mice^30,31^, we detected a moderately higher cyst release in the BALB/c line compared with C57BL/6 mice.

The work of several groups has demonstrated that Th17 responses are pivotal for the control of murine *Giardia* infections^11–13^. Furthermore, a retrospective survey of disease course and memory responses of human patients after clearance of *G. lamblia* infection revealed that individuals that had more readily controlled giardiasis comprised more IL-17A-producing cells in peripheral blood^10^. In line with these reports, we show here that C57BL/6 mice harboring relatively high frequencies of small intestinal Th17 cells together with ILC3 contributing to IL-17A production at steady state and upon infection, controlled *G. muris* infection more efficiently compared to BALB/c mice. Increased cyst shedding in BALB/c mice was not associated with signs of morbidity such as weight loss or small intestinal inflammation, confirming previous reports on minimal inflammation induced by *Giardia* infection^32,33^. Of note, stronger Th17 activity detected in C57BL/6 mice correlated with mild signs of enteritis in these mice, albeit this was not altered upon *G. muris* infection.

Comparing the two mouse lines at steady state and two weeks (or 7 days, data not shown) post infection, we detected no increase in intestinal IL-17A transcript levels and found stable frequencies of IL-17A producing Th17 cells and ILC3 in the small intestine and PP. However, previous studies clearly demonstrated elevated IL-17A mRNA levels in small intestinal tissue as early as 7 days post *G. muris* infection in C57BL/6 mice, coinciding with more prominent IL-17A expression by small intestinal CD4^+^ T cells and innate immune cells^11,12^. Whether this discrepancy relates to the different origin of experimental animals and their microbiota composition is not clear. Of note, the stable frequencies of IL-17A^+^ Th17 detected in the small intetsines of naïve and infected C57BL/6 mice reported here exceed the levels found in the study by Dann & colleagues^11^, which may have masked a more subtle rise in the activity of intestinal Th17 cells in infected mice in our study.

Contrasting the unaltered local IL-17A expression, we detected elevated proliferation and fatty acid uptake to occur selectively in small intestinal Th17 cells from infected C57BL/6 mice, indicative of more active Th17 cells in these mice. Whether these comprise early stage RORγt^+^ effector cells recently attracted to the infected gut, yet producing low levels of IL-17A due to the paucity of signals educating them for effector cytokine expression^34^, remains to be established. Alternatively, Th17 cells present in higher frequencies in naïve C67BL/6 compared to BALB/c mice may receive signals sufficient for local expansion, but insufficient for the launch of the full effector program. According to our data, BALB/c mice appear even more reluctant in that respect, since these lack any sign of elevated Th17 activity within two weeks of *Giardia* infection. Furthermore, the basis for stable frequencies of small intestinal Th17 cells detected along with increased Th17 cell proliferation in infected C57BL/6 mice reported here deserves further investigation. The similar expression of CD103 and CD69 important for T cell retention in peripheral tissues^35,36^ by small intestinal Th17 cells from naïve and infected mice paired with similar frequencies of systemic Th17 cells in spleen (data not shown) does not support the idea that small intestinal Th17 cells of infected C57BL/6 mice might more readily recirculate from gut tissue. On the other hand, we detected *Giardia*-specific IL-17A production by spleen cells, but not mLN and PP cells of infected C57BL/6 mice controlling parasite replication more efficiently. This finding might reflect the release of highly functional parasite-specific cells from the gut or associated lymphoid tissues into systemic circulation. Whether the apparent lack of parasite-specific responses in PP and mLN of both mouse lines results from the effective suppression of cytokine production by local regulatory populations, such as Treg, remains to be addressed.

We found that elevated RORγt^+^ Treg to Th17 ratios were associated with poor IL-17A production by Th17 cells and ILC3 and correlated with the poor proliferation and metabolic activity of intestinal Th17 cells in infected BALB/c mice. Foxp3^+^RORγt^+^ cells represent a stable Treg subset enriched in the intestinal lamina propria and potently suppress Th17, Th1 and Th2-associated inflammatory programs^20,22,25,37^. It is hence conceivable that the elevated proportions of RORγt^+^ Treg contributed to impaired parasite control in BALB/c mice. In support of this interpretation, a previous study demonstrated that mice selectively lacking the Foxp3^+^RORγt^+^ Treg subset develop elevated intestinal IL-17A responses at steady state and experience exacerbated immunopathology in a chemically-induced colitis model^20^. The relative paucity of RORγt^+^ Treg detected in C57BL/6 mice might hence be linked to the mild signs of inflammation detected in this mouse strain irrespective of *Giardia* infection. Along those lines, RORγt^+^ Treg expressing high levels of IL-10^22,24^ might be important players restricting microbial dysbiosis and gut inflammation recently reported to develop in *Giardia* infected mice in the absence of IL-10^38^.

The generation of RORγt^+^ Treg depends on the presence of gut microbes, with clostridial species, but also other members of Firmicutes, Bacteroidetes and Proteobacteria supporting their differentiation^20,22,25^. Recent work in C57BL/6 mice has demonstrated that infection with *G. lamblia* leads to shifts in the composition of the commensal microbiota along the entire gastrointestinal tract, despite the predominant localization of trophozoites to the proximal small intestine^4,39^. Barash and colleagues detected an enrichment of Proteobacteria paralleled by the depletion of members of the Firmicutes (Clostridiales) in the small, as well as large intestine within two weeks of *G. lamblia* infection^39^. We hence surveyed the abundance of microbial taxa assigned with the ability to expand Treg, such as Lactobacilli, Bifidobacteria and Clostridia^40–42^ via 16S rRNA-based qPCR and found no differences depending on the mouse strain or infection status. However, high-throughput Illumina sequencing revealed that the overall microbiota structure of BALB/c, but not C57BL/6 mice, was significantly altered upon *G. muris* infection and that the clostridia *Intestinimonas*, *Ruminiclostridium* 5 and *Blautia* moderately decreased in infected BALB/c mice. Infected C57BL/6 mice also displayed moderate changes in the abundances of Clostridia members, namely an increase of *Clostridiaceae* 1 and *Blautia*. Whether the subtle changes between naïve and infected mice concerning the composition of Clostridiales relate to the distinct RORγt^+^ Treg to Th17 cell ratios detected in our study and provide the basis for the distinct phenotypical profiles of small intestinal Treg awaits further investigation.

Gut microbes have been extensively shown to modulate inflammatory and regulatory mucosal immune responses^28,43^ and are likely to affect innate and adaptive anti-*Giardia* immune responses. The generation and accumulation of Th17 cells found in the intestine at steady state depends to a large extent on the microbial colonization of the gut and is potently induced by SFB primarily colonizing the ileum^44,45^. Here, we observed similar abundances of SFB in naïve BALB/c and C57BL/6 mice, however an earlier study provided evidence for the impact of the microbiome composition and, possibly, SFB colonization on *Giardia* control. Singer & Nash showed that C57BL/6 mice provided by two vendors (later shown to differ in SFB colonization^44^), differed in susceptibility to *Giardia* infection^46^. More recently, Paerewijck and colleagues demonstrated that neonate mice infected with *G. muris* displayed delayed IL-17A responses and parasite control^47^. As SFB colonization and the accumulation of intestinal Th17 cells typically occurs in mice at the time of weaning^48^, the delayed control of *G. muris* seen after neonatal infection suggests that Th17 responses triggered by these and other microbes later in life provide an environment equipped for the partial control of *Giardia* infections. Of note, comparably poor Th17 responses of BALB/c mice to gut microbes were shown to result from minor IL-1β production by small intestinal dendritic cells, translating to poor production of serum amyloid A (SAA) by epithelial cells and the more restrained activity of Th17 cells compared to C57BL/6 mice^45^. Hence, the activation of microbe-specific Th17 cells may precede the activity of *Giardia*-specific Th17 cells and this circuit might restrict *Giardia* replication more efficiently in infected C57BL/6 mice already equipped with a more prominent intestinal Th17 cell population. Whether the increased susceptibility of BALb/c mice to *Giardia* infection is is related to their poor IL-1β expression in response to microbes attaching to the gut epithelium^45^, to the higher proportions of RORγt^+^ Treg efficiently restricting the activation of T cells or to peculiarities in their gut microbiota will be assessed in future studies.

Our finding of a significant expansion of SFB in *G. muris*-infected BALB/c, but not C57BL/6 mice displaying higher frequencies of IL-17A producing Th17 cells might point to the preferential activity of regulatory circuits in *Giardia* infected mice. As the degree of SFB colonization is regulated by IL-17A and IgA^49,50^ it will be interesting to assess if *Giardia* infection further stunts Th17 and IgA responses directed against gut microbes in BALB/c mice, permitting the expansion of species colonizing the epithelial layer. Of note, two recent reports demonstrated that *Giardia* infection promotes the secretion of antimicrobial factors in C57BL/6 mice coinfected with *C. rodentium* or *E. coli*, which attenuates pathology induced by bacterial attachment to the epithelial layer^51,52^. Whether such effects are less pronounced on the BALB/c background is not assessed yet.

Finally, in addition to the distinct Th17 profile, naïve and infected C57BL/6 mice displayed significantly more intestinal IgA^+^ plasma cells, higher serum IgA and IgG2b levels, a trend for elevated pIgR expression along the small intestine as well as slightly higher IgA secretion by duodenal tissue explants compared with BALB/c mice. Elevated IgG2b next to IgA levels have been reported previously for *G. muris* infected C57BL/6 mice^53^. The increase in serum IgG2b levels in infected C57BL/6 mice may reflect the more rapid immune activation detected as based on *Giardia*-specifc IL-17A production. Our attempts to detect *Giardia*-specific antibody responses were not successful. However, the lack of parasite-specific IgA and IgG2b in sera after two weeks of *Giardia* infection is in line with a previous study detecting parasite-specific IgA responses no earlier than three weeks post-infection^54^. Furthermore, stable total IgA levels in intestinal secretions reported within four weeks post-infection for *G. muris*-infected BALB/c corroborate our finding of relatively stable IgA levels in both serum and intestinal secretions of BALB/c and C57BL/6 mice at two weeks post-infection. Taken together, the data presented here suggest that the differential susceptibility detected for C57BL/6 and BALB/c mice early after *G. muris* infection are most likely independent of differences in parasite-specific antibody production. Naturally, this does not exclude a differential humoral response developing on different genetic backgrounds may affect the control of *Giardia* at later stages of the infection.

In conclusion, despite discrepancies concerning the local responses of Th17 and innate cells seen in our study compared with previous reports, our finding of distinct patterns of general and parasite-specific Th17 activity linked to the differential susceptibility of two host types supports the established concept of Th17 activity being important for the timely control of *G. muris* infection. Furthermore, our study reveals that *G. muris* infection is associated with more prominent shifts in the microbiota composition in BALB/c mice experiencing higher parasite loads, which includes the thriving of SFB sharing the epithelial niche with *Giardia* trophozoites. The mechanisms deployed by RORγt^+^ Tregs possibly influencing Th17 cell activity during giardiasis and the interplay of microbial signatures and anti-*Giardia* immune responses would therefore merit more in-depth investigations. Furthermore, it will be interesting to see if deficits in microbes supporting homeostatic Th17 responses might be linked to poor control of giardiasis seen in human patients and to investigate which factors support the thriving of potentially pathogenic microbes probably fuelling the symptoms of intestinal irritation observed in symptomatic giardiasis patients.

## Materials and Methods

### Mice and *G. muris* infections

Female BALB/c and C57BL/6 wild-type mice were purchased from Janvier Labs (Saint-Berthevin, France) and maintained under standard specific pathogen-free conditions. All animal experiments were performed in accordance with the National Animal Protection Guidelines and approved by the German Animal Ethics Committee for the Protection of Animals (G0113/15, H0438/17). *G. muris* cysts were originally purchased from Waterborne, Inc. (USA) and were later maintained by passage in BALB/c mice. Cysts were isolated from fresh fecal samples via a sucrose gradient, as described previously^55^ and were administered to mice via oral gavage at a dosage of 1,000 cysts per mouse suspended in 200 μl distilled water. After two weeks, *G. muris*-infected mice and age/sex-matched naïve controls were sacrificed by isoflurane inhalation, followed by cervical dislocation. For faecal cyst counts, 3–4 faecal pellets were collected individually from each mouse 3 times a week, followed by weighing and homogenization in 2 mL distilled water. Cyst numbers were counted in a Neubauer hemocytometer chamber and for weekly cyst shedding over the course of a 6 week infection period as presented in Fig. 1a, the mean value of cyst shedding was calculated across the three collection time points per week.

### Preparation of single cell suspensions

Spleens, Peyer’s patches and mesenteric lymph nodes were isolated and kept in cold RPMI 1640 medium, containing 1% FCS, 100 U/ml penicillin and 100 μg/ml streptomycin (all from PAA, Pasching, Austria). To prepare single cell suspensions, PP were pre-digested in medium with 0.1 mg/ml liberase TL (Roche, Basel, Switzerland) and 0.1 mg/ml DNase (Sigma-Aldrich, St. Louis, MO, USA) at 37 °C on a shaker for 30 min. Spleen, PP and mLN tissues were then forced through 70 μm cell strainers (BD Biosciences, Heidelberg, Germany). Cell numbers were determined using an automated CASY cell counter (Roche-Innovatis, Reutlingen, Germany). Small intestinal lamina propria cells were isolated as previously described^56^.

### Flow cytometry

The following antibodies were used for the detection of surface and intracellular markers: CD4 (clone RM4–5), CD69, (clone H1.2F3), CD90.2 (clone 30H12), CD103 (clone 2E7), Foxp3 (clone FJK-16s), RORγt (clone Q31-378), Ki-67 (clone SolA15), Helios (clone 22F6), IL-17A (clone TC11-18H10), CD45R/B220 (clones RA3-6B2 and RA3-B2), CD19 (clone ID3), IgA (clone mA-6E1), IgM (clone eB121-15F9), CD11b (clone M1/70), CD11c (clone HL3), CD3e (clone 145-2C11), Nkp46 (clone 29A1.4), CCR6 (clone 29-2L17). Non-specific binding was prevented by addition of CD16/CD32 blocking reagent (clone 2.4 G). All antibodies were purchased from eBioscience and Biolegend. Dead cells were excluded using eFluor780 fixable viability dye (Thermo Fisher, Waltham, USA). For intracellular staining of cytokines and transcription factors, cells were fixed and permeabilized using the Fixation/Permeabilization kit and Permeabilization buffer from ThermoFisher/eBioscience. Bodipy FLC16 (ThermoFisher/Invitrogen) labeling was performed at 1 μM in PBS containing 1% FCS for 20 min at 37 °C. Samples were analyzed on a Canto II flow cytometer (BD Biosciences, Heidelberg, Germany) and the data was analyzed using FlowJo software Version 10 (Tree star Inc., Ashland, OR, USA).

### Quantitative Real-Time PCR (qRT-PCR)

At necropsy, 0.5 cm tissue snips were excised from duodenal, jejunal and ileal sections of the small intestinal tract. RNA was isolated using the innuPREP RNA kit according to the manufacturer’s instructions (Analytic Jena, Germany). 2 μg of RNA was then reverse-transcribed to cDNA using a High Capacity RNA-to-cDNA kit (Applied Biosystems, Foster City, CA, USA). Relative gene expression was determined via quantitative real-time PCR (qRT-PCR) using 10 ng of cDNA and FastStart Universal SYBR Green Master Mix (Roche). Primer pairs used for gene amplification are as follows: GUSB (F 5′-GCTCGGGCAAATTCCTTTC-3′; R 5′-CTGAGGTAGCACAATGCCCA-3′), IL-17A (F 5′-ACTACCTCAACCGTTCCACG-3′; R 5′-TTCCCTCCGCATTGACACAG-3′), pIgR (F 5′-ACCAATGGTGACTCTCGCTG-3′; R 5′-AGGTTTGGCTCCCTTGTAGC-3′) and TGF-β (F 5′-CTGCTGACCCCCACTGATAC-3′; R 5′-AGGAGACGGAATACAGGGCT-3′). Efficiencies for each primer pair were determined by generating a standard curve, mRNA expression was normalized to the housekeeping gene β-glucuronidase (GUSB) and was calculated by the Roche Light Cycler 480 software.

### *In vitro* stimulation of splenocytes with *Giardia lamblia* trophozoite antigen

For assessment of parasite-specific IL-17A responses, 5 × 10^5^ splenocytes from naïve and *G. muris*-infected BALB/c and C57BL/6 mice were plated on a round-bottom 96-well cell culture plate in 300 uL RPMI 1640 medium, containing 10% FCS, 100 U/ml penicillin and 100 μg/ml streptomycin (all from PAA, Pasching, Austria). The splenocytes were stimulated with either anti-CD3/CD28 antibodies (1ug/mL), *Giardia lamblia* GS strain trophozoite antigen/GS Ag (20ug/mL) or WB6 strain trophozoite antigen/WB6 Ag (20ug/mL). The cells were then incubated for 96 h at 37 °C and 5% CO_2_. After 96 h, the cell culture supernatants were collected and used for measurement of IL-17 via ELISA using the Mouse IL-17 (homodimer) uncoated ELISA kit from Invitrogen according to the manufacturer’s protocol.

### Preparation of intestinal tissue explants for antibody ELISA

At necropsy, 2 cm of tissue from the duodenum, jejunum and ileum sections of the small intestinal tract of naïve and infected BALB/c and C57BL/6 mice were excised and opened longitudinally. Each tissue sample was placed in a separate well in 1 mL pre-warmed RPMI 1640 medium, containing 1% FCS, 200 U/ml penicillin and 200 μg/ml streptomycin (all from PAA, Pasching, Austria) on a 48-well plate. The intestinal tissue explants were cultured for 24 h at 37 °C and 5% CO_2_. Following incubation, the supernatants were collected and stored at −20 °C for further analysis via ELISA.

### Antibody detection by enzyme-linked immunosorbent assay (ELISA)

Total IgA and IgG2b antibody titres in blood serum and intestinal tissue explant supernatants were quantified via sandwich ELISA. Briefly, 96-well flat bottom Maxisorp plates (Thermo Fischer Scientific, MA, USA) were coated with 50 μL goat anti-mouse IgA (Southern Biotech, AL, USA) and incubated overnight at 4 °C. Plates were washed using a Tecan Hydrospeed microplate washer and blocked with 200 μL 3% BSA in PBS for 1 h before 50 μL samples and standards were added. Plates were incubated with samples and standards for 2 h at room temperature, after which 50 μL goat anti-mouse AP-coupled IgA detection antibody (Southern Biotech, AL, USA) was added for 1 h. 50 μL phosphatase substrate was then added and plates were incubated for 30 min at 37 °C. To stop the enzymatic reaction, 25 μL 100 mM EDTA was added and the signal was measured at 405 nm minus reference wavelength 630 nm on a Biotek Synergy H1 Hybrid Reader.

### Histopathological scoring and immunohistology

Formalin-fixed and paraffin-embedded 1-2 um sections of duodenum, jejunum and ileum were de-waxed and stained with hematoxylin and eosin (H&E) for overview and with fluorescently labelled anti-CD4 and anti-Foxp3 antibodies for the detection of T cells. CD4^+^ and CD4^+^Foxp3^+^ cells were counted along five villi per section. Enteritis was scored using H&E-stained sections as described before^57^. All evaluations were performed blind.

### Analysis of fecal microbiota via 16S rRNA-based qPCR

Fresh fecal pellets collected from naïve controls and mice infected with *G. muris* for two weeks were immediately snap-frozen in liquid nitrogen and stored at −80 °C until further processing. DNA was extracted from fecal samples using the innuPrep Stool DNA kit (AnalyticJena) as per the manufacturer’s instructions. For the qPCR analysis in brief, DNA was quantified by using Quant-iT PicoGreen reagent (Invitrogen, UK) and adjusted to 1 ng/μL. Then, the main bacterial groups abundant in the murine intestinal microbiota including Enterobacteria, Enterococci, Lactobacilli, Bifidobacteria, *Bacteroides/Prevotella* spp., Mouse Intestinal Bacteroides, *Clostridium coccoides* group, and *Clostridium leptum* group, as well as total Eubacterial loads were assessed by quantitative real-time PCR (qRT-PCR) with species-, genera-, or group-specific 16S rRNA gene primers (Tib MolBiol, Germany) as described previously^58^. Numbers of 16S rRNA gene copies per nanogram DNA of each sample were determined.

### High-throughput bacterial 16S rRNA Illumina sequencing of faecal microbiota

Genomic DNA was extracted from a total of 25 faecal samples collected form infected and uninfected mice of each line (i.e. 8 infected and 4 uninfected BALB/c, and 7 infected and 6 uninfected C57BL/6) as described above. High-throughput sequencing of prokaryotic 16S rRNA gene was performed on an Illumina MiSeq platform. Briefly, the V3-V4 region was PCR-amplified using universal primers^59^, that contained the Illumina adapter overhang nucleotide sequences, using the Q5^®^ NEBNext hot start high-fidelity DNA polymerase (New England Biolabs), 5 ng/μL of template DNA and the following thermocycling protocol: 2 min at 98 °C, 20 cycles of 15 s at 98 °C – 30 s at 63 °C – 30 s at 72 °C, and a final elongation step of 5 min at 72 °C. Amplicons were purified using AMPure XP beads (Beckman Coulter) and set up for the index PCR using Q5^®^ NEBNext hot start high-fidelity DNA polymerase and Nextera XT index primers (Illumina), with thermocycling as follows: 3 min at 95 °C, 8 cycles of 30 s at 95 °C – 30 s at 55 °C – 30 s at 72 °C, and 5 min at 72 °C. The indexed samples were purified using AMPure XP beads, quantified using the Qubit dsDNA high sensitivity kit (Life Technologies), and equal amounts from each sample pooled. The resulting pooled library was quantified using the NEBNext library quantification kit (New England Biolabs) and sequenced using the v3 chemistry (2 × 300 bp paired-end reads, Illumina). The raw sequences are available from Mendeley (DOI: 10.17632/sj5cms7fky.1).

### Statistical analysis and bioinformatics

Statistical analysis of FACS data was performed using GraphPad Prism software (La Jolla, CA, USA). Results are displayed as mean ± SD and significance is displayed as *p < 0.05, **p < 0.01, ***p < 0.001. Results were tested for normal distribution using the Shapiro-Wilk normality tests, followed by ANOVA or Kruskal-Wallis combined with Tukey’s or Dunn’s multiple comparison testing. For the correlation analysis between RORγt^+^ Treg:Th17 ratios, FLC16^+^ Th17 and Ki-67^+^ Th17 cell frequencies, Spearman’s rank correlation coefficient was calculated.

Paired-end demultiplexed Illumina reads were processed using the Quantitative Insights Into Microbial Ecology (QIIME2; 2019.4 release) software suite^60^. Sequences were then quality filtered, dereplicated, chimeras were identified, and paired-end reads were merged in QIIME2 using DADA2^61^ with default settings. A phylogenetic tree was generated using the align-to-tree-mafft-fasttree pipeline in the q2-phylogeny plugin. Bray-Curtis dissimilarity between samples was calculated using core-metrics-phylogenetic method from the q2-diversity plugin. Classification of Operational Taxonomic Units (OTUs) was performed using a Naïve Bayes algorithm trained using sequences representing the bacterial V3-V4 rRNA region available from the SILVA database (; Silva_132)^62^, and the corresponding taxonomic classifications were obtained using the q2-feature-classifier plugin in QIIME2. The classifier was then used to assign taxonomic information to representative sequences of each OTU.

Statistical analyses were executed using the Calypso software^63^ (cgenome.net/calypso/). For data normalisation, cumulative-sum scaling (CSS) was applied to the OTU table, followed by log2 transformation (CSS + log) to account for the non-normal distribution of taxonomic counts data. Samples from each mouse line were ordinated using unsupervised PCoA based on Bray-Curtis dissimilarities, and supervised CCA was then performed including infection status as explanatory variable. The same analyses were applied to samples from naïve BALB/c and C57BL/6 mice in order to address the differences in gut microbial community structure between both lines at the steady state. Following rarefaction of raw data, differences in microbial alpha diversity (Shannon diversity), richness and evenness were evaluated using ANOVA. Beta diversity was calculated using Bray-Curtis dissimilarity and differences in beta diversity were calculated using Analysis of Similarity (ANOSIM)^64^. Differences in the composition of the microbiota between groups were assessed using the LEfSe workflow^65^ on CSS + log transformed data, as well as negative binomial distribution (DESeq. 2)^66^, the latter applied on not normalised, not rarefied datasets. Relative abundances were calculated on count data normalised by the total sum scaling (TSS) normalization method.

## Supplementary information

Supplementary Information

## Data Availability

The datasets used and analyzed in the current study are available from the corresponding author on reasonable request. The raw 16S rRNA data are available at Mendeley Data (doi: 10.17632/sj5cms7fky.1).
