# Supplementary material for: RORγt+ Treg to Th17 ratios correlate with susceptibility to Giardia infection
Source: Sci Rep. 2019 Dec 30;9:20328. doi: 10.1038/s41598-019-56416-9 (PMC6937251; doi:10.1038/s41598-019-56416-9)
Supplement: Supplementary file 1 — Supplementary Information [file 41598_2019_56416_MOESM1_ESM.docx]

RORγt^+^ Treg to Th17 ratios correlate with susceptibility to *Giardia* infection

Ivet A. Yordanova, Alba Cortés, Christian Klotz, Anja A. Kühl, Markus M. Heimesaat, Cinzia Cantacessi, Susanne Hartmann, Sebastian Rausch

**Supplementary information**

**Supplementary Figure 1**

**Supplementary Figure 1.** **Tissue retention markers on siLP Th17 cells, histological survey of CD4^+^ T cell/Treg counts and Treg phenotypes in naïve and *G. muris* infected** **BALB/c and C57BL/6 mice.**

(**a**) Mean fluorescence intensity (MFI) of the T cell activation/tissue retention marker CD69 as detected for siLP Th17 cells and, as internal controls, CD4^+^Foxp3^-^RORγt^-^ cells from spleen. (**b**) MFI of the tissue retention marker CD103 as described in (a). (**c**) CD4^+^ T cell counts per 5 high power fields (hpf) in small intestinal cross sections. **(d)** CD4^+^Foxp3^+^ T cell counts in small intestinal cross sections. (**e**) Exemplary FACS plots of RORγt and HELIOS expression by small intestinal Foxp3^+^ Tregs. Frequencies of HELIOS^-^ Tregs detected within the total Foxp3^+^ populations of naïve and *G. muris*-infected mice are reported in the bar graph. (**f**) Geometric mean fluorescence intensity (MFI) of Bodipy FLC16 label as detected in Foxp3^+^RORγt^-^, Foxp3^+^RORγt^+^ and Foxp3^-^RORγt^-^ cells isolated from siLP of *G. muris*-infected BALB/c and C57BL/6 mice. a and b: representative for three independent experiments with 2-5/group; c/d: pooled from two independent experiments with n=3-4/group; e/f: pooled from two-three independent experiment with n=3-5/group. Statistical analysis was done using Kruskal-Wallis test combined with Dunn’s multiple comparison test. * p<0.05, ** p<0.01, *** p<0.001.

**Supplementary Figure 2**


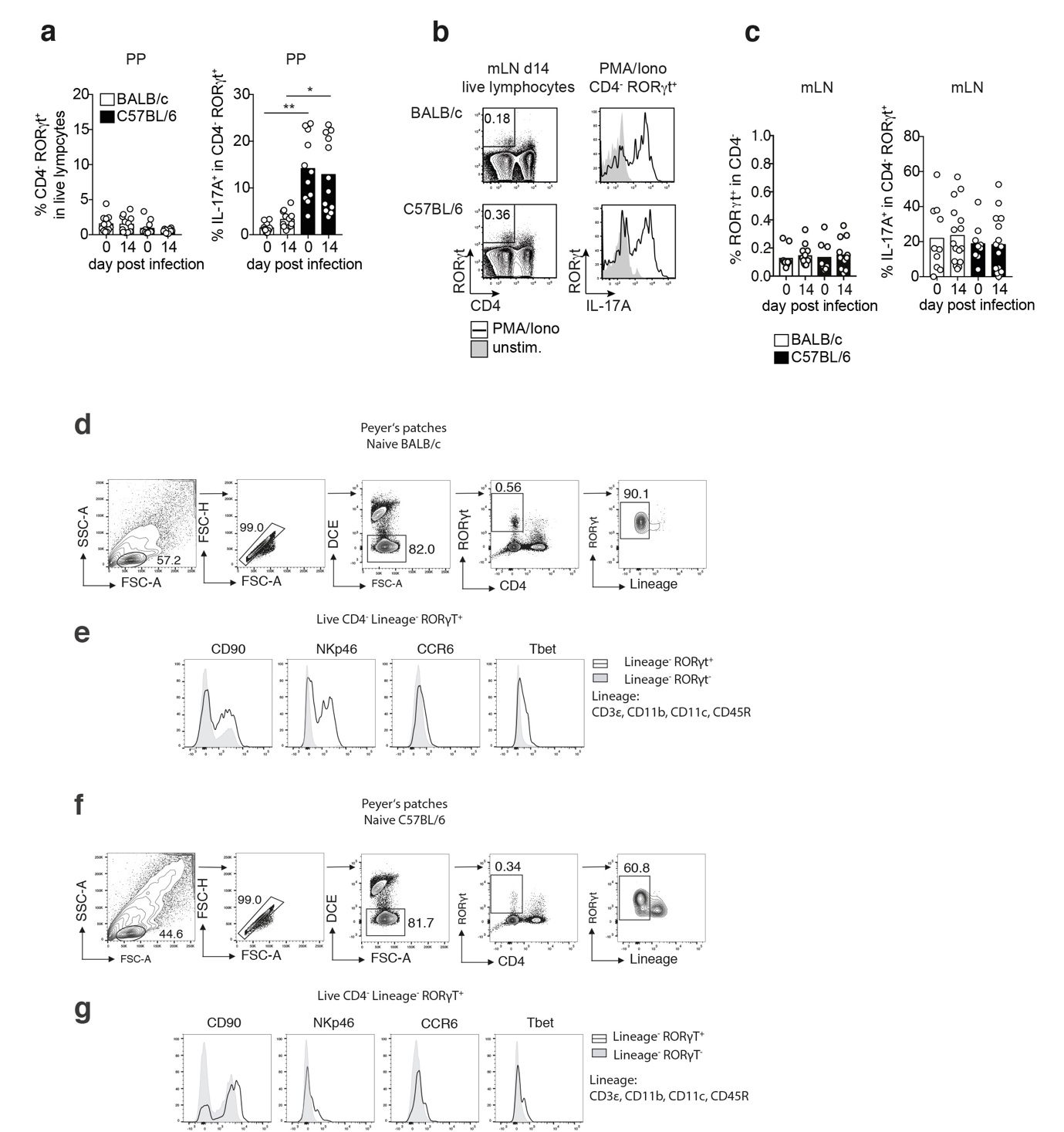


**Supplementary Figure 2. Frequencies of CD4^-^RORγt^+^** **ILC3 cells during *G. muris* infection.**

(**a**) Frequencies of CD4^-^RORγt^+^ cells and their PMA/ionomycin-induced IL-17A responses in cell isolates from Peyer’s patches (PP) of naïve and *G. muris*-infected BALB/c and C57BL/6 mice. (**b)** Left: Representative plots depict the gating of CD4^-^RORγt^+^ cells for mLN cells from BALB/c and C57BL/6 mice at day 14 post *G. muris* infection. Right: IL-17A expression by CD4^-^RORγt^+^ cells from mLN after PMA/ionomycin stimulation. **(c)** Frequencies of CD4^-^RORγt^+^ cells and their PMA/ionomycin-induced IL-17A responses in cell isolates from mesenteric lymph nodes (mLN) of naïve and *G. muris*-infected BALB/c and C57BL/6 mice. **(d, e)** Representative plots depicting (d) the gating strategy for ILC3 in Peyer’s patch cells and **(e)** the expression of CD90, NKp46, CCR6 and T-bet in CD4^-^RORγt^+^ cells in ILC3 of a naive BALB/c mouse. **(f, g)** Representative plots for gating of ILC3 and ILC3 phenotype in a naïve C57BL/6 mouse. Data in a/c are pooled from three independent experiments with n=3-5/group. Statistical analysis was done using Kruskal-Wallis test combined with Dunn’s multiple comparison test. * p<0.05, ** p<0.01, *** p<0.001.

**Supplementary Figure 3**


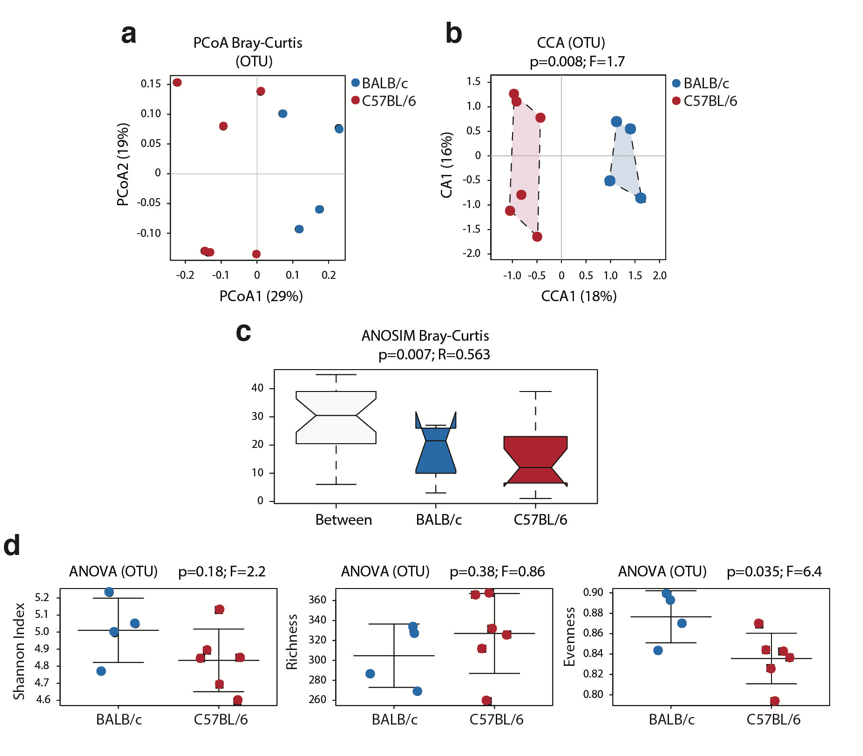


**Supplementary Figure 3. Baseline microbiota differs significantly between BALB/c and C57BL/6 mice.**

**(a, b)** Faecal microbial profiles of naïve BALB/c and C57BL/6 mice ordinated by (a) Principal Coordinates Analysis (PCoA) and (b) Canonical Correspondence Analysis (CCA). (**c**) Microbial beta diversity and in naïve BALB/C and C5BL/6 mice. (**d**) Microbial alpha diversity in naïve BALB/C and C5BL/6 mice. Data are pooled from 2 independent experiments with n=2-4/group.

**Supplementary Figure 4**

**
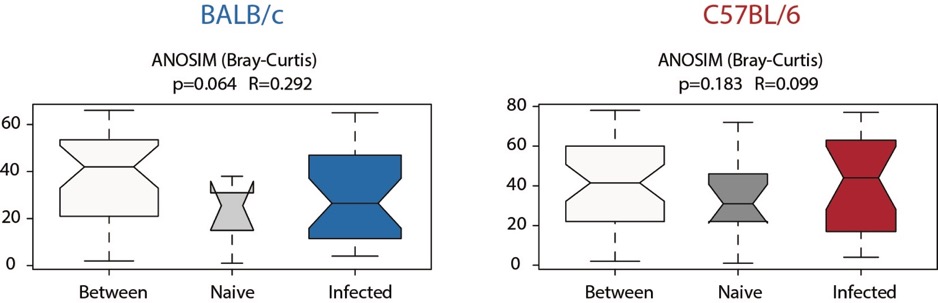
**

**Supplementary Figure 4. Microbial beta diversity is not affected by *Giardia* *muris* infection in either BALB/c or C57BL/6 mice.**

Microbial beta diversity in faecal samples of naïve and *G. muris*-infected mice of each line. Data are pooled from 2 independent experiments with n=2-4/group.

**Supplementary Table 1.** Differentially abundant microbial taxa in the faecal microbiota of BALB/c and C57BL/6 mice under steady state (naïve), based on Linear discriminant analysis Effect Size (LEfSe).

**Supplementary Table 2.** Statistical results for significant differences in the abundance of gut microbial taxa (p<0.05) between BALB/c and C57BL/6 mice under steady state (i.e. naïve) using DESeq2.

**Supplementary Table 3.** Statistical results for significant differences in the abundance of gut microbial taxa (p<0.05) between naïve and infected BALB/c (top) and C57BL/6 mice (bottom) using DESeq2.


**Supplementary Table 4.** Differentially abundant microbial taxa in the fecal microbiota of naive and infected BALB/c and C57BL/6 mice, based on Linear discriminant analysis Effect Size (LEfSe).
